# Effect of cholesterol-loaded cyclodextrin treatment on boar sperm cryopreservation

**DOI:** 10.5713/ab.24.0030

**Published:** 2024-05-07

**Authors:** Silong Zhang, Hanbing Zhang, Kexiong Liu, Xiaoling Xu, Yusheng Qin, Linli Xiao, Chunmei Zhou, Jianliang Wu, Yan Liu, Jiahua Bai

**Affiliations:** 1 Institute of Animal Husbandry and Veterinary Medicine, Beijing Academy of Agriculture and Forestry Sciences, Beijing 100097, China; 2 Beijing University of Agricultural, College of Animal Science and Technology, Beijing 100096, China; 3 Beijing Feifan Biotechnology Co., Ltd., Beijing 100094, China; 4 Beijing Zhongyu Pig Breeding Co., Ltd., Beijing 100194, China

**Keywords:** Boar, Cholesterol, Cyclodextrin, Membrane Structure, Oxidative Damage, Sperm Total Motility

## Abstract

**Objective:**

This study investigated the efficacy of different concentrations of cholesterol-loaded cyclodextrin (CLC) on cryopreservation in boar sperm quality.

**Methods:**

In this study, we treated boar sperm with different concentrations of CLC before freezing and analyzed the sperm cholesterol concentration, plasma membrane, acrosome integrity rate and total motility rate before and after freeze-thawing. We also investigated the levels of reactive oxygen species (ROS), malondialdehyde (MDA), adenosine triphosphate (ATP), and structural- and oxidative-damage related proteins in all groups after thawing.

**Results:**

The results revealed that the cholesterol concentration of the CLC-treated groups was higher than that of the control group, both before freezing and after thawing (p<0.05). The plasma membrane integrity rate, acrosome integrity rate, and total motility rate of sperm were also enhanced after thawing in the CLC-treated group (all p<0.05). Moreover, ROS and MDA production and ATP loss were reduced in CLC-treated sperm during freezing and thawing (p<0.05). Finally, CLC pretreatment partially prevented the consumption of various proteins involved in metabolism including capping actin protein of muscle Z-line subunit beta (CAPZB), heat shock protein 90 alpha family class A member 1 (HSP90AA1) and phosphoglycerate mutase 2 (PGAM2) (p<0.05).

**Conclusion:**

The CLC treatment increased cholesterol concentration and decreased structural injury and oxidative damage during boar sperm freezing and thawing, improving the efficacy of sperm cryopreservation in boar.

## INTRODUCTION

Sperm cryopreservation for artificial insemination (AI) has been widely used for several species of animals, such as bovine, ovine, and fowl, but its application is limited in the pig industry because compared to other animals boar sperm is extremely vulnerable to the “cold shock” experienced during the cryopreservation process [1]. To address this problem, many groups have made efforts to explore the freezing of boar sperm including the freezing procedure and the cryoprotectants. For example, adding seminal plasma from good freezer boars before freezing improved the cryosurvival and *in vitro* fertilizing capacity post thaw [2]. Many antioxidants, such as: superoxide dismutase (SOD), glutathione (GSH), astaxanthin and resveratrol, have a positive effect on boar sperm cryopreservation [3–6]. The above studies focused on the addition of proteins and antioxidants, but the consumption of cholesterol during sperm freezing should also be taken seriously.

Cholesterol has multiple effects on plasma membrane properties, including the order of the acyl chains of phospholipids, stabilization of membranes, and reduction of membrane channels permeability, thus influencing phase transition and the microenvironment for membrane proteins [7]. Cholesterol also affects mitochondrial function, and tyrosine phosphorylation during capacitation, and plays a role in maintaining sperm structure, function, and high fertilization ability [8]. During the freezing process, cholesterol exerts a protective effect on the sperm because it prevents the lateral separation of phospholipids to maintain the integrity and permeability of membrane [9]. The Cholesterol to phospholipid molar ratio, however, is lower in boar sperm membranes (0.26) than that of other animals (0.30, 0.36, and 0.45 in rooster, stallion and bull sperm, respectively), thus making boar sperm more susceptible to membrane structure damage and more vulnerable to freezing injury [10].

Cyclodextrin is a small non-toxic and harmless oligosaccharide with a hydrophobic center and a hydrophilic surface; it is used in coating and as a carrier of drugs and food [11,12]. Methyl-β-cyclodextrin (MBCD) binds to cholesterol and fatty acids, transports them in and out of the cell membrane through a concentration gradient, and inserts cholesterol into the plasma membrane [12,13]. Cholesterol-loaded cyclodextrin (CLC) has been shown to increase sperm cholesterol concentration in ram and bull, leading to improvement in the ability of their sperm to withstand freezing injury and maintain membrane and acrosome integrity, thus enhancing sperm quality for AI [9,14]. In bulls, the abundance of antioxidant enzymes including catalase, glutathione peroxidase, and SOD in CLC-treated sperm were significantly less than the untreated during freezing. Previous studies reported that CLC treatment had a positive effect during cryopreservation of boar sperm, which showed higher percentages of sperm viability and capacitated acrosome-intact after thawing [15–20], but the effects on membrane fluidity and oxidative damage need to be further studied.

In the present study, we investigated the effect of CLC treatment on boar sperm during cryopreservation. We mixed different concentrations of CLC with boar sperm before freezing to detect changes, such as cholesterol concentration, plasma membrane integrity, total motility, and acrosome integrity rate, in sperm before and after freezing. The levels of reactive oxygen species (ROS), malondialdehyde (MDA), adenosine triphosphate (ATP), and freezing-related proteins in thawed sperms were determined to elucidate the underlying reason for the effect of CLC as a cryoprotectant.

## MATERIALS AND METHODS

### Cholesterol-loaded cyclodextrin preparation

The CLC was prepared as described previously [14,21]. Briefly, cyclodextrin solution (1 g of MBCD and 2 mL of methanol) and cholesterol solution (200 mg of cholesterol and 1 mL of chloroform) were prepared separately. A 0.45-mL portion of the cholesterol solution was mixed with the cyclodextrin solution. A stream of nitrogen gas was then passed over the solution and the solvents were allowed to evaporate to obtain a white CLC powder. The CLC stock solution was prepared by dissolving 50 mg of CLC powder in 1 mL of Tris-citric acid-glucose (TCG) buffer (300 mM Tris base, 95 mM citric acid monohydrate, and 28 mM glucose, pH 7.0). The solution was held in a water bath at 39°C for 10 min and then vortexed and sonicated for 30 min. Finally, 3 mg/mL bovine serum albumin was added to obtain a working CLC solution.

### Semen collection and processing

Six adult Yorkshire boars with proven fertility, aged 1.5 to 2.5 years, were selected from a commercial farm (Beijing Zhongyu Pig Breeding Co., Ltd., Beijing, China). All 6 boars were utilized for sperm collection using the hand-glove method and filtered through double layers of sterile cotton gauze to remove the gelatinous portion. The semen from two boars was pooled for subsequent treatment in each repetition to mitigate individual variations, and the experiment was replicated three times. The boar semen used in this study was light, milky white or grayish white, with a specific odor, but no malodor; the sperm total motility was >85%; and the morphologically normal sperm was >90%. The semen was diluted (1:1 v/v) with extender III (Androstar Premium Minitube, Diefenbach, Germany) and transported to the laboratory within 1 h at 17°C. All the experiments were carried out according to the International Guiding Principles for Biomedical Research Involving Animals, and the respective permit was granted by Beijing Academy of Agriculture and Forestry Sciences (IHVM11-1903-1, approved on March 12, 2019).

### Cryopreservation and thawing of semen

The semen was stored at 17°C after arriving at the laboratory [22], the sperm density test (NucleoCounter; Chemometec, City, Denmark) and dilution (to 1.5 to 2×10^8^ sperm/mL) were completed within 20 minutes. The diluted semen was randomly divided into control group (0 mg CLC/1.2×10^8^ sperm, treated with TCG buffer), low-CLC group (0.5 mg CLC/1.2×10^8^ sperm), and high-CLC group (1.0 mg CLC/1.2 ×10^8^ sperm) [20]. The sperm and CLC were mixed and incubated at 17°C for 20 min (according to our preliminary data shown in Table 1), then the semen was centrifuged at 700×*g* for 9 min. Pellets were recovered and diluted at 1.5 ×10^9^ sperm/mL in freezing extender I (Androstar Cryo Plus containing 20% egg yolk; Minitube, Germany). Spermatozoa were cooled to 5°C for 120 min and subsequently diluted at 1.0×10^9^ sperm/mL in freezing extender II (Androstar Cryo Plus containing 20% egg yolk,15% glycerol; Minitube, Germany) [22–24]. The final concentration of semen was 1.0×10^9^ sperm/mL, 20% egg yolk and 5% glycerol. The processed semen was loaded into 0.5 mL straws (Minitub, Germany), and sealed with polyvinyl chloride powder before being placed in contact with nitrogen vapor approximately 3 cm above the liquid nitrogen level for 15 min. Then, straws were plunged into liquid nitrogen (–196°C). The straws were then stored in liquid nitrogen until thawing.

After one week of freezing, straws of frozen semen were thawed at 50°C for 16 s in the water bath. The thawed semen was then extended in extender III (Androstar Premium; Minitube, Germany) at a final ratio of 1:10 to give a concentration of 1.0×10^8^ sperm/mL, and incubated at 38°C for 15 min before further analyses [25].

### Semen analysis

The assessments of sperm total motility, cholesterol concentration, plasma membrane integrity, and acrosome integrity rate were made in pre-freeze (after equilibration) and thawed sperm. All tests contained three biological replicates per group.

### Assessment of sperm total motility

The sperm total motility and the normal morphology rate were evaluated using the CASA system (Sperm Class Analyzer CASAS-QH-III; Tsinghua Tongfang, Beijing, China), with standard parameter settings as previously described [26]. Briefly, after incubation at 37°C for 15min, 4μL of each sample was loaded into a 37°C preheated chamber (one-time sperm counting slides; Yufan, Yancheng, China) and warmed at 37°C for 30s before microscopic examination. Subsequently, three visual fields, containing at least 300 spermatozoa, were randomly selected to record the total motility data and the sperm morphology in every sample.

### Analysis of total cholesterol concentration

The amount of cholesterol in each sample was determined using the cholesterol Assay Kit (Solarbio Inc., Beijing, China) as previously described [27]. Briefly, the semen samples were washed with phosphate buffered saline (PBS) three times, followed by the addition of isopropanol to the sperm precipitate. The mixture was ultrasonicated in an ice-bath, incubated at 4°C or ice for 5 to 10 min, and centrifuged at 12,000×*g* for 10 min. The supernatant was collected, and the absorbance of each supernatant was quantified three times using a spectrophotometer (Infinitem Plex Tecan, Män nedorf, Switzerland) at a wavelength of 500 nm.

### Sperm plasma membrane and acrosome integrity

Similarly to previous studies, the evaluation of sperm membrane integrity was carried out by the association of fluorescent probes (propidium iodide [PI] and Hoechst 33342) [21]. In brief, each 100μL of semen was co-incubated with 10μL of a 12μM PI and 10 μL of a 1 μg/mL Hoechst33342 at 38°C for 10min. Then, the semen was fixed with 4% paraformaldehyde for 10 min and observed under a fluorescence inverted microscope (Olympus CH 30, RF-200; Olympus, Tokyo, Japan). Sperm with an intact plasma membrane emitted blue fluorescence, while spermatozoa with pink fluorescence had a disrupted plasma membrane and the cells were considered to be dead. The staining characteristics were evaluated for at least 200 spermatozoa/microscopic field to assess plasma membrane integrity.

The acrosome integrity was determined by co-staining of spermatozoa with lectin from Arachis hypogaea (peanut agglutinin) conjugated with fluorescein isothiocyanate (FITC-PNA) and PI. In short, a 50μL aliquot of semen was applied to a glass slide and fixed in anhydrous methanol for 15min at room temperature. Fifty microliters each of 10μg/mL PI solution and 100μg/mL FITC-PNA solution were sequentially added to each slide with a 10min interval between additions, and the slide was placed in a wet box at 37°C for 30min. Slides were then rinsed three times with PBS and air-dried. A fluorescence inverted microscope was used to analyze the percentages of acrosomal intact sperm. For each sample, the staining of at least 200 spermatozoa was evaluated.

### Detection of reactive oxygen species levels

The intracellular ROS levels were measured using a reactive oxygen species assay Kit (Beyotime, Shanghai, China) as described by by Min et al [28] and Li et al [29]. Briefly, the sperm samples (1.2×10^8^ sperm/mL), containing three biological replicates in each group, were incubated with DCFH-DA (10μM) at 38°C in a darkened area for 25min, then centrifuged at 1,500×*g* for 4min, and the pellet was rinsed twice with PBS. The pellet was subsequently re-suspended in PBS and the ROS concentrations were measured at excitation/emission wavelengths of 488/525nm three times by a fluorescence spectrophotometer. The changes of sperm ROS were analyzed by comparing the ratio of fluorescence value to sperm density in each group.

### Sperm malondialdehyde, adenosine triphosphate, and bicinchoninic acid concentrations analysis

The kits, included the lipid oxidation (MDA) Detection Kit, ATP Detection Kit, and bicinchoninic acid **(**BCA) Protein Concentration Determination Kit, were purchased from Beyotime (China). The samples were ultrasonicated at 4°C for 5 to 10 min followed by centrifuging at 12,000×*g* for 10 min, and the upper layer was collected for further testing. For MDA assay, the MDA detection solution was mixed with standard solution (1, 2, 5, 10, 20, 50 μM) and test samples. Then, the mixes were incubated at 100°C for 15 min, followed by cooling to room temperature. The MDA levels were measured by a fluorescence spectrophotometer at 532 nm. For BCA test, the BCA detection solution was mixed with standard solution (0, 0.025, 0.05, 0.1, 0.2, 0.3, 0.4, 0.5 mg/mL) and test samples. Then, the mixes were incubated at 37°C for 25 min. The BCA levels were measured by a fluorescence spectrophotometer at 595 nm. About ATP content test, the ATP detection solution was mixed with standard solution (0.01, 0.03, 0.1, 0.3, 1, 3, 10 μM) and test samples. The ATP detection solution was added to the detect-well to eliminate background ATP, followed by adding the test samples to the detect-well for measuring ATP contents by luminescence rapidly.

### Western blotting

Three semen samples in each group were lysed with RIPA (Beyotime, China) containing 1 mM PMSF and phosphatase inhibitor (Beyotime, China). Protein concentration was detected by the BCA method. Semen lysates (15 μg) were separated by 12% sodium dodecyl sulfate-polyacrylamide gel electrophoresis (SDS-PAGE) and transferred onto polyvinylidene fluoride (PVDF) membranes (200 mA, 90 min). The membranes were blocked with blocking solution (Beyotime, China) and incubated with primary antibodies (Table 2) overnight at 4°C followed by washing three times using tris buffered saline with tween 20 (TBST). The membrane was incubated with horseradish peroxidase-conjugated secondary antibodies for 2 h at room temperature, whereafter, washing three times using TBST. The α-tubulin protein was used as a loading reference. The membranes were treated with super ECL Plus and the western blotting signals were detected by a gel imaging system (Bio-Rad, Hercules, CA, USA). Every protein was detected using different PVDF membranes. The intensities of blotting signals were quantified by outlining the relevant bands on the film with the Image-Lab software.

### Statistical analysis

Statistical analyses were conducted using the SPSS software version 26.0 (SPSS, Inc., Chicago, IL, USA). First, data were evaluated for normality (Shapiro-Wilk’s test) and homogeneity of variance (Levene's test). Data, including total cholesterol concentration, acrosome integrity, plasma membrane integrity, sperm morphology and sperm total motility before freezing and after thawing and ROS concentrations, MDA content, ATP levels and protein expression levels after thawing were analyzed using the one-way analysis of variance, followed by use of the least significant difference post-hoc test. The figures were drawn using GraphPad Prism software version 9.0 (GraphPad software; San Diego, CA, USA). Results are presented as mean±standard deviation, and p<0.05 was considered to indicate a significant difference.

## RESULTS

### Effect of cholesterol-loaded cyclodextrin on the quality of boar sperm before freezing

**Figure 1:**
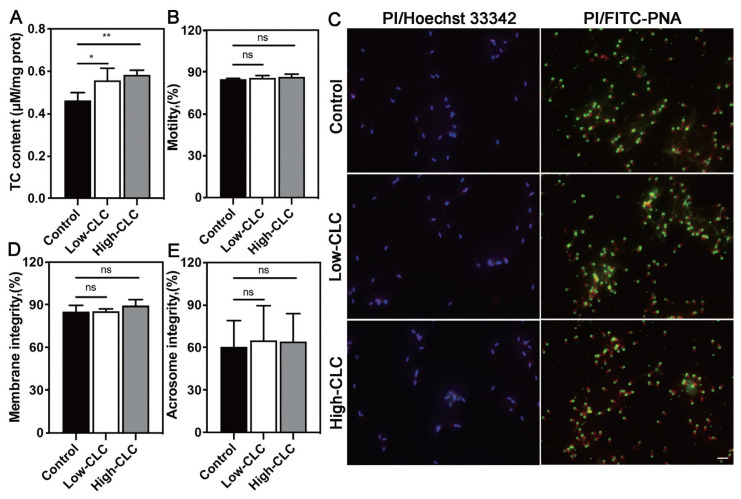
Effect of CLC-treatment on boar sperm before freezing. The TC content (A) and total motility of boar sperm before freezing (B). The representative images of membrane and acrosome integrity staining determined in boar sperm before freezing, scale bar = 20 μm (C). The statistics of membrane integrity, and acrosome integrity of boar sperm before freezing (D and E). Values are presented as mean±standard deviation (n = 3). CLC, cholesterol-loaded cyclodextrin; TC, total cholesterol. Control, no CLC treatment; low-CLC, 0.5 mg CLC/1.2×10^8^ sperm; high-CLC, 1.0 mg CLC/1.2×10^8^ sperm. * p<0.05, ** p<0.01. ns, no significant difference.

The total cholesterol (TC) concentration, total motility, membrane integrity and acrosome integrity of boar sperm before freezing are shown in Figure 1. No surprisingly, the result showed that CLC treatment significantly increased the TC concentration before freezing (Figure 1A; p<0.05). However, we did not observe a significant difference in total motility (Figure 1B). In addition, we detected the membrane integrity and acrosome integrity of boar sperm by using fluorescence staining. As shown in Figure 1C, the staining results revealed that the number of PI^+^ sperm (left) and the acrosome intact sperm (right) have no difference among the three groups, the quantitative results are displayed in Figure 1D–1E. There was no difference in the sperm morphology treatment without and with different CLC concentrations before freezing (p> 0.05; Table 3).

### Effect of cholesterol-loaded cyclodextrin on the quality of frozen-thawed boar sperm

**Figure 2:**
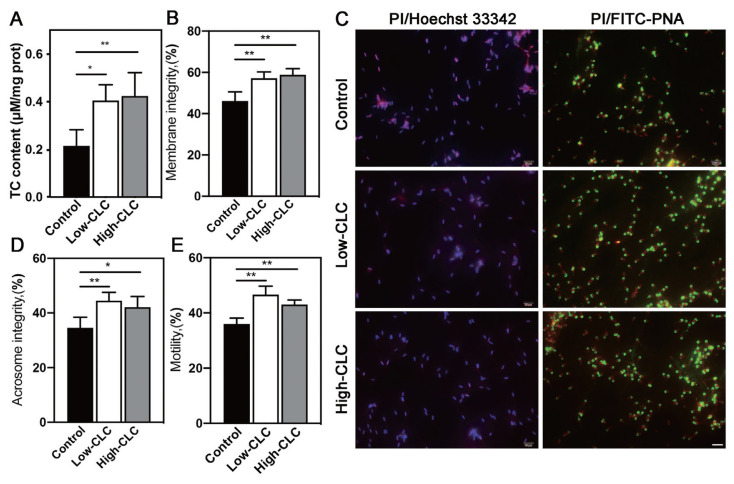
Effect of CLC-treatment on frozen-thawed boar sperm. The TC content of frozen-thawed boar sperm (A). The representative images of membrane and acrosome integrity staining determined in frozen-thawed boar sperm, scale bar = 20 μm (C). The statistics of membrane integrity, acrosome integrity (B and D) and the total motility (E) of frozen-thawed boar sperm. Values are presented as mean±standard deviation (n = 3). CLC, cholesterol-loaded cyclodextrin; TC, total cholesterol. Control, no CLC treatment; low-CLC, 0.5 mg CLC/1.2×10^8^ sperm; high-CLC: 1.0 mg CLC/1.2×10^8^ sperm. * p<0.05, ** p<0.01.

Based on the increase in TC concentration before freezing, we investigated the TC concentration, total motility, membrane integrity and acrosome integrity after thawing. CLC treatment, similarly, significantly increased the TC concentration after thawing (p<0.05; Figure 2A). Then the staining result showed that the number of PI^+^ sperm (left) and the acrosome intact sperm (right) in the two CLC treatment groups were higher than the control group (Figure 2C), the quantitative results displayed in Figure 1B and 1D (p<0.05), respectively. Further, the total motility of the frozen-thawed sperm was significantly enhanced by CLC treatment (p<0.01; Figure 2E), however, post-thaw sperm morphology was not improved after CLC treatment (p>0.05; Table 3).

### Effects of cholesterol-loaded cyclodextrin treatment on reactive oxygen species, malondialdehyde, and adenosine triphosphate levels of frozen-thawed sperm

**Figure 3:**
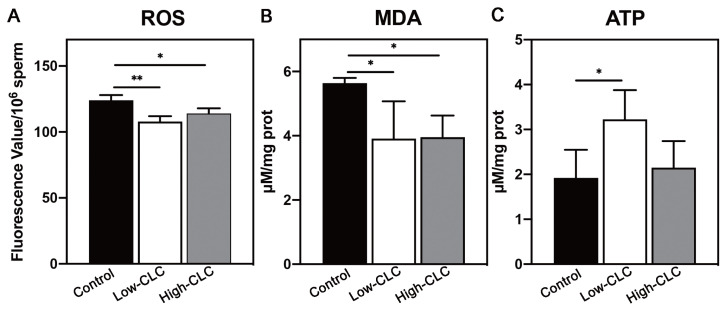
The levels of ROS (A), MDA (B), ATP (C) in frozen-thawed boar sperm treated with different concentrations of CLC. Values are presented as mean±standard deviation (n = 3). ROS, reactive oxygen species; MDA, malondialdehyde; ATP, adenosine triphosphate; CLC, cholesterol-loaded cyclodextrin. Control, no CLC treatment; low-CLC, 0.5 mg CLC/1.2×10^8^ sperm; high-CLC, 1.0 mg CLC/1.2×10^8^ sperm. * p<0.05, ** p<0.01.

To elucidate the underlying reason for the CLC-induced improvement in sperm quality, we evaluated ROS, MDA, and ATP concentrations as indicators of sperm oxidative damage and metabolism during the freeze-thawing process. The CLC treatment significantly reduced the ROS level of frozen-thawed sperm (p<0.05; Figure 3A). Meanwhile, the concentration of MDA, a harmful oxidative metabolite, was lower in the CLC-treated groups than the control group (p<0.05; Figure 3B). Only the low-CLC group, however, exhibited higher ATP level than the control group (p<0.05; Figure 3C).

### Effect of cholesterol-loaded cyclodextrin treatment on protein consumption in frozen-thawed sperm

**Figure 4:**
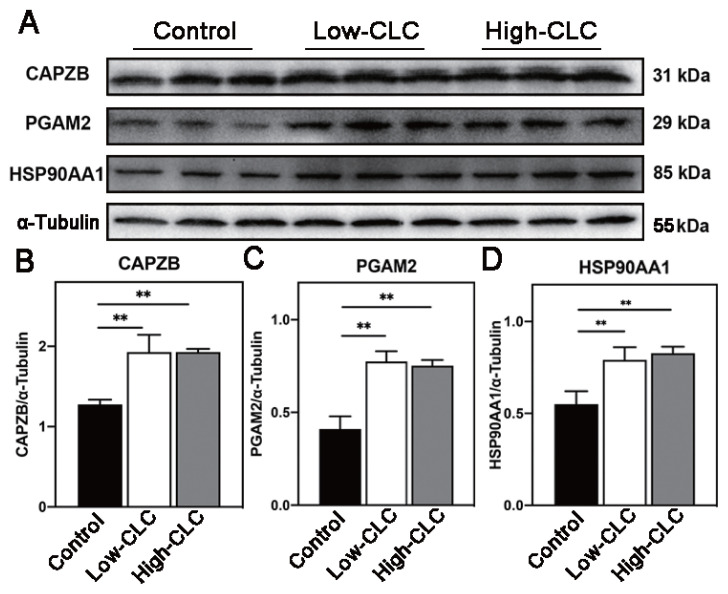
Effect of CLC-treatment on sperm protein consumption during freezing and thawing. The protein levels of CAPZB, PGAM2, and HSP90AA1 detected by western blotting (A). The quantification of CAPZB, PGAM2, and HSP90AA1 abundance detected by gray scanning (B–D). Values are presented as mean±standard deviation (n = 3). CLC, cholesterol-loaded cyclodextrin; CAPZB, capping actin protein of muscle Z-line subunit beta; PGAM2, phosphoglycerate mutase 2; HSP90AA1, heat shock protein 90 alpha family class A member 1. Control, no CLC treatment; low-CLC, 0.5 mg CLC/1.2×10^8^ sperm; high-CLC, 1.0 mg CLC/1.2×10^8^ sperm. ** p<0.01.

To further understand the mechanism of CLC-treated improvement in sperm quality and oxidative damage, we investigated the abundance of proteins related to sperm structural integrity, metabolism, and stress. Figure 4 shows that the protein levels of capping actin protein of muscle Z-line subunit beta (CAPZB), phosphoglycerate mutase 2 (PGAM 2), and heat shock protein 90 alpha family class A member 1 (HSP90AA1) were significantly higher in CLC-treated groups than in the control group (p<0.01; Figure 4).

## DISCUSSION

In this study, we pretreated boar sperm with CLC to enhance the efficacy of cryopreservation. Our results showed that CLC pretreatment increased the TC concentration of sperm thereby improving membrane and acrosome structure integrity, meanwhile, mitigating the oxidative stress and energy loss caused by freezing.

During freezing, cold shock causes a series of changes in the physical and chemical properties of sperm, including membrane fluidity reduction, aggravation of oxidation reaction, and decreased sperm membrane integrity and acrosome integrity. Among these changes, the sperm membrane structure is the most vulnerable to damage during freezing [16]. Sperm with low cholesterol concentration, such as boar sperm, are more sensitive to temperature changes and more vulnerable to structural damage and oxidative damage caused by low temperature [19]. Previous studies have reported that transport of cholesterol into sperm via cyclodextrin could improve sperm viability after freeze-thawing in stallion, ram, and bull [30–33]. Similar to the results reported for stallion and boar sperm [19,34], in this study, although cholesterol levels losses were found in each group after freezing, higher cholesterol concentration of boar sperm treated with CLC was observed than sperm untreated before freezing and after thawing, indicating that cholesterol consumption occurs during freezing but CLC could effectively place cholesterol into the sperm. Meanwhile, significant improvements in total motility, membrane integrity and acrosome integrity after thawing were observed in CLC-treated than in untreated sperm, but not before freezing. This suggests that cholesterol supplementation contributed to the improvement of total sperm total motility, membrane integrity and acrosomal integrity in frozen-thawed sperm.

Previous studies have found that CLC treatment can improve the quality of freeze-thawed boar sperm by increasing sperm membrane cholesterol content [35]. Oxidative injury, however, also is a major factor affecting the preservation of sperm during freezing [36]. Reactive oxygen species are a by-product of oxidative phosphorylation via mitochondrial metabolism, and MDA is the end product of sperm lipid oxidation [33]. Reactive oxygen species, present in high levels, react with sulfhydryl groups in phospholipids, oxidases, and other proteins, and induce the cleavage of DNA molecules, resulting in sperm cell membrane damage and sperm motility decline [37]. The results of the present study revealed that the ROS levels in the CLC-treatment groups decreased, and MDA concentration was also significantly lower than that of the control group. This is consistent with our previous inference that CLC-pretreatment of boar sperm could reduce oxidative damage due to freezing, which may be one of the reasons for the improved sperm quality. Our results are also validated in similar studies in ram and jack donkey that indicated CLC can reduce the oxidative damage of sperm caused by low temperature [32,38].

It is currently generally believed that ATP generated in the process of glycolysis is very important for boar sperm viability during liquid storage [39,40]. A decrease in sperm motility also occurs after inhibition of either the mitochondrial respiratory chain complex I or oxidative phosphorylation, suggesting that mitochondria-transformed energy also has an important function in maintaining boar sperm metabolism [41]. In rats, adding ATP to thawing buffer has a beneficial role in maintaining the function of frozen-thawed rat sperm [42]. In the present study, CLC had a pronounced effect on mitigating the ATP level decline of the sperm during freezing, indicating CLC could allow for sustaining normal mitochondrial function in boar sperm cryopreservation. Moreover, increase in ROS levels decreases ATP production and destroys the mitochondrial transcription system, thus affects the quality of sperm, which further confirms the reliability of ATP results through the decrease of ROS levels in CLC treatment groups [43].

CAPZB is a skeleton protein involved in stabilizing the sperm skeleton network, sperm capacitation, and acrosome reaction [44]. Under normal conditions, the CAPZB content is highly positively correlated with sperm motility [45]. Based on the enhancement of sperm quality observed after CLC treatment, we investigated the abundance of CAPZB to evaluate the degree of structural damage in frozen-thawed sperm. The results revealed that CLC treatment reduced the loss of CAPZB protein in boar sperm during freezing. This further elucidated the protective effect of CLC treatment on the structural integrity of boar sperm during the freezing process. PGAM2, an essential enzyme, participates in the glycolytic pathway, and its content reflects the state of the tricarboxylic acid cycle [46]. Wojtusik et al [47] reported that CLC treatment of gazelle sperm reduced the effect of freezing on sperm glycolysis by reducing the degradation of PGAM2. We found that the PGAM2 levels in CLC pretreatment sperm were higher than control group after thawing, suggesting CLCs had a rescuing effect on PGAM2 during freezing and thawing of boar sperm; helping to explain increased motility and ATP levels in pretreated boar sperm. HSP90AA1, a molecular chaperone protein, is involved in ATP metabolism and therefore, it is possible that the decrease in motility observed in thawed sperm may be a result of decreased HSPs leading to reduced ATP level [48]. In human, bull and gazelle, cryopreservation possibly caused the degradation of the HSPs protein [47–49]. In this study, following CLC treatment the decrease of HSP90AA1 was drastically less than control group. Our results suggest that CLC minimizes the loss of HSP90AA1, possibly promoting rescue of motility in sperm when treated with CLC. Collectively, CLC treatment significantly decreased the consumption of CAPZB, PGAM2, and HSP90AA1 in boar sperm during freezing and thawing, which guarantees the function of sperm after thawing. The three proteins play an important role in sperm structure and function including sperm skeleton network and ATP metabolism, meanwhile, reflected by our sperm structure and ATP content assay results.

## CONCLUSION

We did not observe significant difference in sperm quality between the low-CLC group and the high-CLC group, but treatment with low concentration of CLC exhibited greater potential for protection of boar sperm against freezing injury, which was evident by the ATP concentration of thawed sperm. Therefore, pretreatment of boar sperm with 0.5 mg CLC/1.2×10^8^ sperm may enable better preservation of sperm against the damage caused due to cryopreservation. And positive effects of CLC treatment were observed in terms of the reduction in structural damage and oxidative damage and improvement in the metabolic state of the freeze-thawed sperm.

## Figures and Tables

**Table 1:** The total motility (%) of frozen-thawed sperm treated with different concentrations CLC and times

Concentration of CLC (mg)	15 min	20 min	30 min
0	12.89±5.17	17.67±11.39	11.73±8.00
0.5	24.64±7.14	30.35±16.17	15.35±5.98
1.0	17.84±9.82	22.80±11.01	15.04±12.48

Results are expressed as mean±standard deviation.

CLC, cholesterol-loaded cyclodextrin.

**Table 2:** Primary antibodies and secondary antibodies used for western blotting

Parameter	Antibody name	Host species	Dilution	Vendor	Code
Primary antibodies	HSP90AA1	Rabbit	1:2,000	Proteintech	13171-1-AP
	PGAM2	Rabbit	1:2,000	Proteintech	15550-1-AP
	CAPZB	Rabbit	1:2,000	Abcam	ab96618
	α-Tubulin	Rabbit	1:1,000	Cell Signaling Technology	2148
Secondary antibody	Goat Anti-Rabbit	Goat	1:4,000	Proteintech	SA00001-2

**Table 3:** Boar sperm morphology before freezing and post-thaw treatment without and with different cholesterol-loaded cyclodextrin concentrations

Items (%)	Before freezing (mg)			Post-thaw (mg)		
	
	0	0.5	1.0	0	0.5	1.0
Deformity rate	17.90±2.33	17.20±1.18	17.34±0.70	19.50±1.14	18.10±1.28	18.34±0.40
Coiled tail	3.95±0.56	4.21±1.24	6.23±1.44	7.94±1.18	6.88±2.09	4.86±1.02
Short tail	8.46±0.32	6.14±2.27	7.42±1.01	7.43±0.64	5.76±2.55	8.86±0.41
Isolated head	5.49±1.68	6.86±2.25	3.70±1.13	4.12±2.20	5.45±1.69	4.62±1.43

Results are expressed as mean±standard deviation.
